# Unveiling the Mutations and Conservation of InlA in *Listeria monocytogenes*

**DOI:** 10.3390/microorganisms12030485

**Published:** 2024-02-28

**Authors:** Lingling Li, Yan Wang, Ji Pu, Jinni Chen, Lingyun Liu, Pan Mao, Hui Sun, Xia Luo, Changyun Ye

**Affiliations:** 1 National Key Laboratory of Intelligent Tracking and Forecasting for Infectious Diseases, National Institute for Communicable Disease Control and Prevention, Chinese Center for Disease Control and Prevention, Beijing 102206, China; ling605782805@163.com (L.L.); wangyan@icdc.cn (Y.W.); puji@icdc.cn (J.P.); 15034624562@163.com (J.C.); llyyyx2021@163.com (L.L.); maopan_7@163.com (P.M.); sunhui@icdc.cn (H.S.); luoxia@icdc.cn (X.L.); 2 The Key and Characteristic Laboratory of Modern Pathogen Biology, School of Basic Medical Sciences, Guizhou Medical University, Guiyang 550004, China

**Keywords:** *Listeria monocytogenes*, InlA, ST, amino acid, protein sequence type

## Abstract

*Listeria monocytogenes* (*L. monocytogenes*) is a pathogen that is transmitted through contaminated food and causes the illness known as listeriosis. The virulence factor InlA plays a crucial role in the invasion of *L. monocytogenes* into the human intestinal epithelium. In addition, InlA enhances the pathogenicity of host strains, and different strains of *L. monocytogenes* contain varying variations of InlA. Our study analyzed a total of 4393 published *L. monocytogenes* genomes from 511 sequence types (STs) of diverse origins. We identified 300 unique InlA protein sequence types (PSTs) and revealed 45 highly mutated amino acid sites. The leucine-rich repeat (LRR) region was found to be the most conserved among the InlA, while the protein A (PA) region experienced the highest mutation rate. Two new types of mutations were identified in the B-repeat region of InlA. Correspondence analysis (CA) was used to analyze correlations between the lineages or 10 most common sequence types (STs) and amino acid (aa) sites. ST8 was strongly correlated with site 192_F, 454_T. ST7 exhibited a strong correlation with site 51_A, 573_E, 648_S, and 664_A, and it was also associated with ST6 and site 544_N, 671_A, 738_B, 739_B, 740_B, and 774_Y. Additionally, a strong correlation between ST1 and site 142_S, 738_N, ST2 and site 2_K, 142_S, 738_N, as well as ST87 and site2_K, 738_N was demonstrated. Our findings contribute significantly to the understanding of the distribution, composition, and conservation of InlA in *L. monocytogenes.* These findings also suggest a potential role of InlA in supporting molecular epidemiological tracing efforts.

## 1. Introduction

*Listeria monocytogenes* (*L. monocytogenes*) is a foodborne pathogen that can cause gastroenteritis in healthy individuals, meningitis in immunocompromised individuals, and may lead to abortions in pregnant women, with a fatality rate of up to 20–30% [1]. *L. monocytogenes* is a Gram-positive saprophytic species that can survive in harsh environments, such as low temperatures, partial sanitizers, a broad range of pH, and high salt levels, among other conditions [2]. As a facultative intracellular parasite, *L. monocytogenes* can invade and survive within a wide range of non-phagocytic cells. 

InlA is the first identified virulence factor linked with *L. monocytogenes* invasion. It can be located on the host cell membrane by binding to the E-cadherin receptor and enhances the crossing of both the intestinal and placental barrier [3]. Point mutations in the InlA may result in premature stop codons (PMSC) and truncated InlA, which makes it a secretory protein and cannot be anchored to the bacterial cell wall. This weakens the ability of *L. monocytogenes* to invade intestinal epithelial cells. Previous research has shown this on Caco-2 epithelial cells [4,5,6,7].

*L. monocytogenes* is categorized into four lineages (I, II, III, and IV), with I and II being the most prevalent, showing noticeable differences in sources [8]. Most of the strains in lineage I are clinical isolates, whereas the lineage II strains are predominantly isolated from contaminated foods [9]. Lineages III and IV strains are less common and obtained mainly from animals. The multi-locus sequence typing (MLST) of *L. monocytogenes* is determined by seven housekeeping genes [10]. The clonal complex (CC) refers to sequence types that vary only in one allele. Studies suggest that CC1, CC2, CC4, and CC6 are the primary clone complexes linked to listeriosis, whereas CC9 and CC121 are common clone complexes of *L. monocytogenes* in food [11,12,13].

The intestinal barrier represents the initial defense against *L. monocytogenes* infection, and InlA is pivotal for enabling the bacteria to invade [14]. A lot of studies have reported that PMSCs in InlA, resulting in a truncated form of InlA, suggesting attenuated pathogenicity [15,16]. For example, in the United States, the detection of *L. monocytogenes* strains harboring stop codons has been demonstrated at 35–45% in food isolates, while human isolates only account for 5.1% [17]. At present, many types of PMSC mutations in the InlA have been documented [18,19,20]. However, Wang et al. showed that the presence of PMSC type 19 at position 976 may not impact the ability of *L. monocytogenes* to adhere to and invade epithelial cells [21]. Moreover, Dellafiora drew attention to missense mutations, which are often overlooked, and suggested that they should be investigated for their potential effects on cell invasion processes [22]. Indeed, there is a lack of comprehensive analysis of InlA amino acid sequence characteristics. Early research on the InlA in *L. monocytogenes* solely focused on the presence or absence of PMSC, using a small number of total genomes or a few STs of interest [23]. Nowadays, thousands of *L. monocytogenes* genomes are available in public databases. We conducted an InlA examination on *L. monocytogenes* utilizing a significantly larger data set than prior studies [9,24,25]. The aim of this study was to investigate the distribution and length of InlA in *L. monocytogenes,* identify the characteristics of InlA protein sequences among strains of different lineages, sources and STs, and obtain more understanding about the role of InlA in causing listeriosis.

## 2. Materials and Methods

### 2.1. Genomes of Listeria monocytogenes

A total of 4393 genomes of *L. monocytogenes* were collected from various sources and diverse geographic locations, including 318 newly sequenced genomes from our laboratory (Chinese Center for Disease Control and Prevention), and 4075 publicly available assembled genomes (available to download until March 2022) from 43 other countries on six continents (Supplementary Table S1). There were 695, 1580, 167, and 1090 strains from humans, food, animals, and the environment respectively, with 861 strains from an unknown source.

### 2.2. MLST Analysis

Using seven housekeeping genes (*abcZ*, *bglA*, *cat*, *dapE*, *dat*, *ldh*, and *lhkA*), the local BLAST method was employed to determine the ST type of the strains. If the MLST of the strain is unknown, the genome sequence is submitted to the Genome Pasteur Database website (https://bigsdb.pasteur.fr/cgi-bin/bigsdb/bigsdb.pl?db=pubmlst_listeria_seqdef&page=sequenceQuery (accessed on 7 September 2023)) for MLST determination (Supplementary Table S1).

### 2.3. Alignment and Analysis of InlA Sequence

MEGA software (version 11) was used to align and obtain amino acid sequences of the InlA. Protein types of InlA were identified by comparing them with the reference strain (EGD-e, NC_003210.1) by using local BLAST methods [20]. An amino acid difference is a new protein sequence type (PST). The presence of an asterisk (∗) in the matched query sequences indicated a PMSC happening in the genome assembly sequences. The mutation sites were identified by BLASTN with the reference sequence, while the specific types of PMSC were referenced from previous studies [18,19,20]. The graphical representation of the amino acid probabilities at each site was generated by WebLogo [26].

### 2.4. Statistical Analysis

The relationship among the *L. monocytogenes* lineages or STs (columns) and the aa of the InlA site (row) (Table S2) was explored by corresponding analysis (CA) in R language [19]. The angle between the row and column arrows represents the correlation between them, and the smaller the angle, the stronger the correlation.

## 3. Results

### 3.1. Distribution Characteristics and MLST of L. monocytogenes Genomes

**Figure 1:**
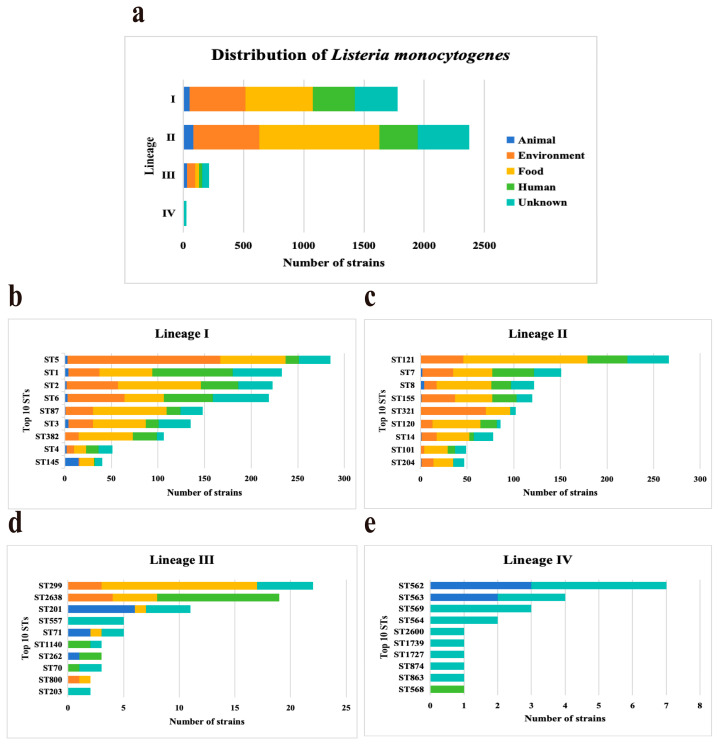
Distribution characteristics and domain sequence types of *Listeria monocytogenes*. (**a**) Distribution of four Lineages (I, II, III, and IV) in five sources. (**b**–**e**) Distribution of top 10 sequence types (STs) in four Lineages among the 4393 *L. monocytogenes* strains.

Four lineages were identified among 4393 *L. monocytogenes* strains, including lineage I (*n*  =  1781, 40.54%), II (*n*  =  2375, 54.06%), III (*n*  =  213, 4.85%), and IV (*n*  =  24, 0.55%). Lineages distributions of *L. monocytogenes* strains were from four sources (Figure 1a). Lineages I, II, and III accounted for a similar proportion of strains from sources. Lineages IV strains were predominant in the unknown with a frequency of 85.71% (18/21). Based on MLST, all the studied isolates were classified into 511 STs and grouped into 290 CCs. ST distributions of *L. monocytogenes* strains were different in four lineages (Figure 1). ST5 (*n*  =  285, 16%), ST121 (*n*  =  266, 11.2%), ST299 (*n*  =  22, 10.33%), and ST562 (*n*  =  7, 29.17%) strains were predominant in Lineages I, Lineages II, Lineages III, and Lineages IV, respectively. All the *L. monocytogenes* strains selected in this study carried the InlA. 

### 3.2. The Classification and Analysis of InlA Sequence Types

In this study, a total of 300 PSTs of InlA were identified from the strains and classified into two groups based on the presence of PMSC: the non-PMSC group (group A) and the PMSC group (group B). Groups A and B were then further subdivided into six (by InlA length) or seven subgroups (by truncated in the domain of InlA). Group A consisted of 262 PSTs, accounting for 77.44% of the total strains (3402/4393), while group B had 38 PSTs, accounting for 22.56% (991/4393). In group A, there were 2995 strains from A-1 (800aa), 351 from A-2 (797aa), 33 from A-3 (730aa), 21 from A-4 (799aa), 1 from A-5 (733aa), and 1 from A-6 (801aa). Among these, the PSTs that encode 800 amino acids exhibited maximum diversity, encompassing 162 types.

**Figure 2:**
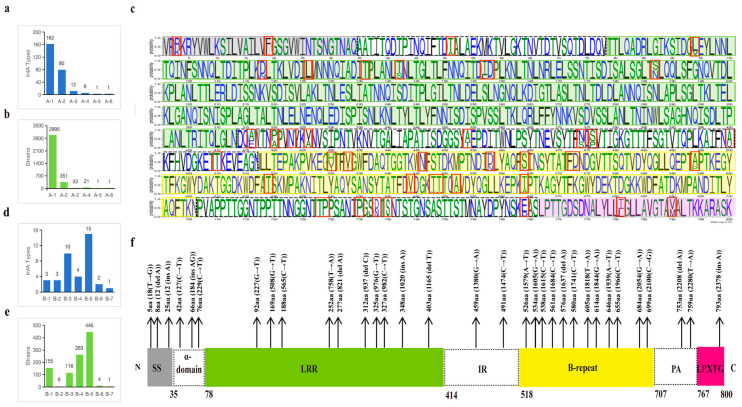
Distribution characteristics of InlA sequences in this study. Distribution of InlA types (**a**) or strains (**b**) in group A. Map of the 261 mutation sites in the protein sequences of group A, where the red boxes mark the sites where more than 10 types of protein sequences are mutated (**c**). Distribution of InlA types (**d**) or strains (**e**) in group B. The length of truncated of InlA and nucleotide position of mutation in group B (**f**).

The InlA sequences of group B were truncated in the signal sequence (SS), α-helix region, Leucine rich repeats (LRR) region, inter-repeat (IR) region, B-repeat region, Protein A (PA) region, and Leu-Pro-X-Thr-Gly C terminal cell wall anchor motif (LPXTG), X for any amino acid region with 3, 3, 10, 2, 12, 2, and 1 truncation types, respectively (Figure 2f). B-5 (B-repeat) exhibited the highest number of strains and the most diverse PSTs in group B. While the number of strains in B-4 (IR) exceeded those in B-3 (LRR), the PSTs of B-3 were noticeably more diverse. Three types of truncations were identified in group B-1 (SS), alongside a greater number of strains (*n* = 155). Similarly, in group B-2 (α-helix), three truncation types were found, but only six strains. B-5 and B-6 each had four and one strains, respectively, and these strains belonged to two or one PSTs, respectively.

**Figure 3:**
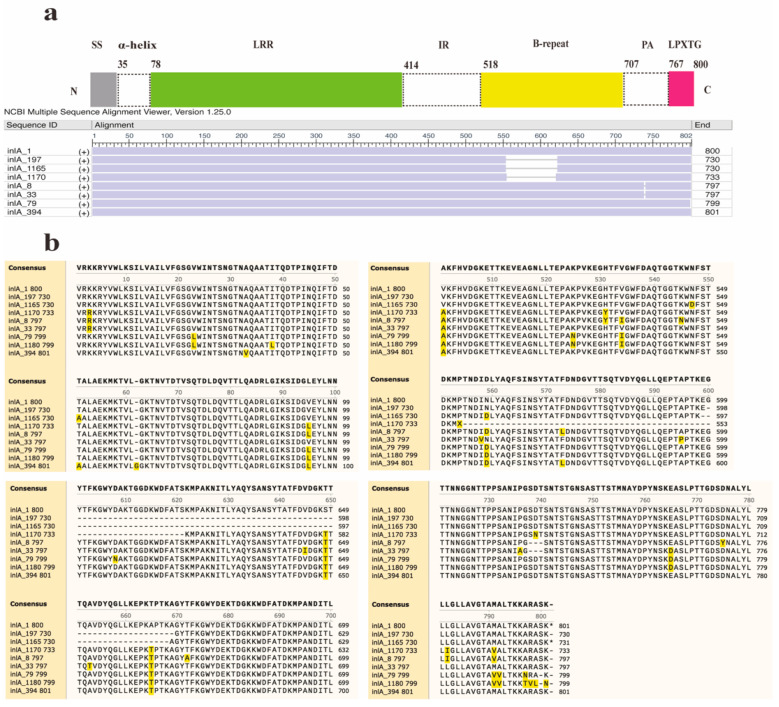
Alignment and schematic representation of the InlA mutant and EGDe (InlA_1). (**a**) Represents the overall schematic diagram of InlA alignment, and (**b**) shows the specific location of the mutation. SS: Signal Sequence.

Furthermore, two new InlA mutations encoding proteins with 730 and 733 amino acids were discovered in this study (Figure 3). Out of the 12 distinct types of PST encoding 730 amino acids, 11 sequences were deficient in 70 amino acids at positions 599–668, whereas another sequence (InlA_1165) had a deficiency of 70 amino acids at positions 598–667. The InlA sequences of group A-3 encode 730 amino acids (Figure 3b). It was found predominantly in lineage II (30/33) strains and was distributed in lineages I and III. The sequence of group A-5 encodes 733 amino acids exhibited a deletion of 67 amino acids at positions 554–621 compared to InlA of EGD-e. All PSTs in Group A-2 encode 797 amino acids and showed a deletion of three amino acids (SDT) at positions 737–739. There is a total of 80 InlA PSTs in Group A-2, predominantly from lineage III strains, but also including lineage I and II strains. The most common protein types in this group were PST 6 (InlA_8) and PST 25 (InlA_33), which also existed in China. Furthermore, Group A-4 results from a deletion of one amino acid (S) at position 799, leading to 799 amino acids. Six PSTs encoding 799 amino acids in group A-4 were solely identified in lineage IV strains. Finally, group A-6 arises from an insertion of a ‘G’ amino acid at position 62, producing 801 amino acids.

**Figure 4:**
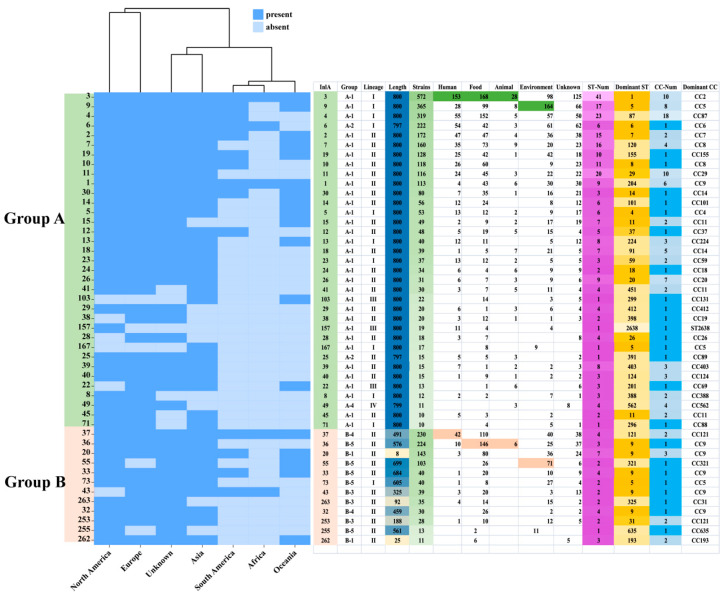
Distribution of 47 InlA types (*n* ≧ 10). The InlA number, the lineage, source, location, ST, and CC are shown on the right rows. The color legend is shown above.

To investigate the relationship between the InlA and the strains, 47 InlA PSTs (*n* ≥ 10, 3964 strains) were selected for further analysis. We found out that most protein types are distributed in multiple continents (Figure 4). However, PST 167 was only found in Asian strains; PST 157, on the other hand, was only found in North American strains. Out of these 47 PSTs, 35 PSTs were grouped into group A, and the most common PSTs detected in humans, food, animals, and environmental sources were PST 3, PST 3, PST 3, and PST 9, respectively. Figure 4 shows that out of 32 PSTs that encode 800 aa, 15 corresponded to a single CC, while the remainder corresponded to multiple CCs. Within group A, PST 3 was the most common corresponding to 10 CCs, with CC2 as the dominant type. PST 9 followed, corresponding to 8 CCs, and CC5 was the dominant type. PST 4 corresponded to 18 CCs, in which CC87 was the most dominant CC type. Group B strains were predominantly concentrated in lineage II, followed by lineage I. Only a single strain belonging to group B-7 was identified in lineage III, which encoded 793 amino acids. Lineage I strains were distributed mainly in groups B-1, B-3, B-5, and B-6. Among them, B-5 (B-repeat) represented the highest proportion, accounting for 78.79% (52/66) of the total. Lineage II strains had a broader distribution ranging from groups B-1 to B-6. Additionally, group B-5 (B-repeat) accounted for the highest proportion again, at 42.64% (394/924). Among groups B-1, B-5, and B-6, CC9 was the most dominant CC type. Figure 4 shows that out of the 12 PSTs (*n* ≥ 10), in group B, 8 PSTs correspond to one CC type while the other 4 PSTs correspond to multiple CC types. PST 37, PST 20, PST 253, and PST 262 correspond to 2, 3, 2, and 2 CC types, respectively, with the dominant CC types of CC121, CC9, CC121, and CC193. 

Group B includes 67 human strains, representing 6 CCs with CC121 predominating (43/67) (Table 1). Among the human strains, 64/67 are from lineage II with the remaining 3 strains belonging to lineage I. Truncated InlA regions were identified in the following distribution: 62.69% (42/67) of the strains in the IR region (B-4), 20.9% (14/67) in the B repeat region (B-5), 11.94% (8/67) in the LRR region (B-3), and 4.48% (3/67) in the SS region (B-1). Additionally, these strains included 9 PMSC types, with the PMSC type 6 (491 aa) being the most prevalent, accounting for 62.69% (42/67). 

### 3.3. The Classification and Analysis of InlA Sequence Types

The 261 PSTs of InlA in Group A (except for the sequence of one strain, 801aa) were compared with the InlA of EGD-e, and further analysis revealed that 526 were fully conserved and 274 were variable sites (Table S1). There were 45 amino acid mutation sites that exhibited a substitution rate higher than 1.47% (*n* > 50, 50/3401). The mutation rates in various structural domains of amino acids, named SS, α-helix, LRR, IR, B-repeat, PA, and LPXTG regions were calculated to be 8.82% (3/34), 9.3% (4/43), 1.79% (6/336), 6.73% (7/104), 8.47% (16/189), 10% (6/60), and 8.82% (3/34), respectively. The highly conserved IR region was best described as an immunoglobulin (Ig) and is structurally the most flexible part of the internalin domain [27]. In our study, the LRR region of InlA exhibited the highest conservation, while the PA region displayed the most frequent mutations, followed by the α-helix region, these two high mutation regions may have no effect on binding receptor function [28].

**Figure 5:**
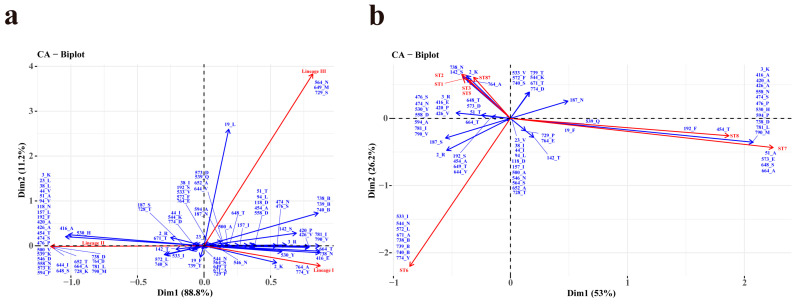
Correspondence analysis (CA) between the aa site (*n* > 50, represented in blue) and lineages (**a**) or ten most prevalent STs (**b**) (represented in red) of *Listeria monocytogenes* strains. “B” represents the deletion of aa.

Certain amino acid sites exhibit specific amino acids, including acidic, basic, polar, or nonpolar residues, and a subset of these sites (*n* > 50) demonstrate a pronounced correlation with lineages (Figure 5a). Specifically, Site 764_A, and 774_Y are significantly associated with lineage I. A minor association between site 2_K and lineage I was also observed. Site 3_K, 23_L, 38_L, 44_V, 51-A, 94_V, 118_N, 157_L, 192_F, 420_A, 426_A, 454_T, 474_S, 476_P, 500_V, 539_K, 546_D, 573_E, 594_P, 644_I, 648_S, 652_T, 664_A, 558-N, 728_K, 738_D, 764_D, 781_L, and 790_M were demonstrated a strong correlation with lineage II. A minor association among site 416_A, 530_H and lineage II was also observed. Site 564_N, 649_M, and 729_S showed a high correlation with lineage III. A minor association between site19_L and lineage III was also observed. On the contrary, lineage IV did not exhibit a particular association with any specific site.

In Figure 5b, the associations among the ten most prevalent STs and aa sites (*n* > 50) were shown using CA in R. The graphical representation shows a strong correlation between ST1 and site 142_S, 738_N, ST2 and site 2_K, 142_S, 738_N, as well as ST87 and site 2_K, 738_N. Correspondingly, ST7 is related to site 51_A, 573_E, 648_S, and 664_A. Moreover, ST8 is strong related to site 192_F and 454_T, and as well as among ST6 and site 544_N, 671_A, 738_B, 739_B, 740_B, and 774_Y. Additionally, the site 3_K, 416_A, 420_A, 426_A, 474_S, 530_H, 558_N, 594_P, 738_D, 781_L, and 790_M demonstrated a minor correlation with ST7 and ST8. In addition, site 738_N and 764_A exhibited a strong correlation with ST3 and ST5. 

A correlation was observed between amino acids at 21 out of 45 sites (sites 2, 3, 19, 23, 38, 44, 94, 142, 157, 187, 474, 500, 533, 564, 572, 644, 648, 739, 740, 781, and 790) and a specific lineage, without any alteration in their polarity or charge (Table S2). The remaining 24 sites exhibited distinct attributes, with 10 sites (site 118, 530, 539, 544, 546, 558, 573, 728, 738, and 774) altering only the charge without affecting the polarity, and 14 sites (site 51, 192, 416, 420, 426, 454, 476, 594, 649, 652, 664, 671, 729, and 764) changing the polarity of the amino acid side chain. Sites 51_A (α-helix), 192_F (LRR), 420_A, 426_A, 454-T, 476_P (IR), 594-P, 649_B, 652_B, 652_T, and 664_A (B-repeat) with changed polarity exhibited a strong correlation with lineage II. Meanwhile, sites 416_E and 764_A had a strong correlation with lineage I, and sites 649_M, 664_M, and 729_S significantly correlated with lineage III. It is worth mentioning that site 420 (*n* = 1) and site 594 (*n* = 1) were both mutated to threonine, resulting in a change from nonpolar amino acids to polar, uncharged amino acids.

## 4. Discussion

A thorough comprehension of the polymorphism, truncation types, and distribution characteristics of Internalin A is crucial for studying and evaluating the virulence of *L. monocytogenes*, as it constitutes a significant virulence factor [29]. Furthermore, advances in whole-genome sequencing technology enable us to analyze the epidemiological features of *L. monocytogenes* strains via the InlA and to investigate their potential virulence by InlA sequences.

In this study, a total of 4393 strains of *L. monocytogenes* were divided into 4 lineages, which were then clustered into 290 CCs. The dominant CCs varied from different sources, with CC1 (99/695), CC9 (310/1580), CC2 (17/167), and CC5 (166/1090) were the dominant CCs for strains obtained from humans, food, animals, and environmental sources, respectively. Studies have shown that CC1 and CC2 are the most prevalent clones in clinical strains, whereas CC9 is often found in food and environmental samples and is a clone with low-virulence [19]. ST5 and ST121 are known to exhibit higher tolerance to adverse conditions and their capacity to adapt to such environments has been found to be crucial in the formation of biofilms, thereby affecting the persistence of *L. monocytogenes* in the environment [30,31,32].

The PST 3 (lineage I, 800 aa) of InlA exhibited the highest carriage rates among strains found in humans (153/659), animals (28/167), and food (168/1580). This suggested that the strains of PST 3 existed in the dominant three sources may be due to the most common PST3 strains. PST 3 corresponded to 10 CCs, with CC1 (269/572) as the dominant type. The PST 9 (lineage I, 800 aa), with the dominant CC type being CC5 (221/365), has the highest carriage rate among environmental strains (164/1090). The correlation between InlA and CC is typically one-to-one with a few exceptions. For example, PST 4 (lineage I, 800 aa) corresponded to 18 CCs, with CC87 as the dominant type. CC87 is made up of ST87 and three other STs, which is the most common subpopulation connected with food, recreational beach sands, and human clinical infections in China [33,34,35]. Among groups B-1 (SS), B-5 (B-repeat), and B-6 (PA), CC9 was the most dominant CC. This observation could be attributed to the mutations that CC9 strains have undergone to adapt to their respective environments, primarily food and the surroundings. Therefore, InlA can serve as a biomarker of virulence for strains.

Based on the alignment of 300 InlA sequences with that of EGD-e, the LRR region was identified as the most conserved, while PA was the most active mutation region. The LRR region is known to play a critical role in InlA binding, while the LPXTG region serves as a membrane anchoring region [36,37]. These two key functional regions are highly conserved, indicating that they were necessary for the InlA. Among the truncated InlA variants, the B repeat region was the most frequently truncated, followed by the IR region, then the SS region, LRR region, α-helix region, PA region, and LPXTG region [35]. There is no necessary link between the frequency of amino acid mutations and the truncation of InlA, which can possibly be attributed to the environmental pressures of coping with complex external factors. This study identified two new InlA mutations, which encode 730 and 733 amino acids, respectively. These mutants do not fall under the PMSC classification. A previous study indicated that knocking down the B repeat or PA regions has no impact on the invasion of *L. monocytogenes* [28], but these findings were limited to artificially constructed InlA mutants. Further confirmation is required for the invasion of naturally occurring truncated InlA with a B repeat region mutation. Our study found that 33 strains from 13 STs harbored 730aa-InlA, including 30 lineage II strains, one lineage I strain, and one lineage III strain, which shows that the 730aa mutation type is dominant in lineage II strains. All of them belong to the US Food and Drug Administration's surveillance project for the rapid detection of foodborne contamination events (PRJNA304956). Twenty-nine strains were collected from Italy and four from the United States. The predominant ST of the Italian strains was ST204 (6/29), while all four US strains (collected from Snow King Peach) belonged to ST1331. Drupes, including peaches, are generally considered to pose a low risk of foodborne illness. However, in 2014, an outbreak involving *L. monocytogenes* highlighted the potential of drupes as a new food vector for transmitting *L. monocytogenes*, based on cases of human listeriosis [38]. Italy faced a continuous outbreak caused by an invasive lineage II strain of *L. monocytogenes* [39,40]. These findings indicate the importance of taking this type of mutation seriously.

Previous research has shown that the InlA, which encodes 797 amino acids, experiences a deletion of 3 amino acids in the C-terminal sequence and still maintains a complete LPXTG region [35]. We discovered that InlA PST 6, which encodes 797 amino acids, is more commonly present among the strains and belongs to lineage I, serotype IVb, which is similar to earlier research [20]. Strains of serotype IVb have been responsible for several listeriosis outbreaks [11]. For instance, in 2017–2018, the outbreak in South Africa was caused by ready-to-eat meat products contaminated with these strains, leading to a widespread infection with a mortality rate of 28.6%. The strains associated with the outbreak were of serotype 4b and ST6 genotype [35,41]. Our research indicates that the InlAs encoding for 797 amino acids exhibited the most diversity in lineage III. The strain had a 3-amino acid deletion in the transmembrane region near the C-terminus of InlA and was accountable for the 2002 outbreak of contaminated ready-to-eat meat. However, this type still retained its virulence [42,43]. Kovacevic and colleagues reported a similar variation in which three amino acids were deleted from a strain isolated from food and food processing environments in British Columbia, Canada. This variation was able to invade Caco-2 cells [43]. Although we did not test the invasion of these InlA proteins, we inferred from their sequence alone that they have invasive capabilities. 

Most *L. monocytogenes* strains belong to Lineages I and II, while Lineages III and IV are infrequent and are primarily found in ruminants [44,45]. Our study discovered that 22.56% (991/4393) strains carried truncated InlA belong to group B, with 93.24% (924/991) of those strains being lineage II. Additionally, InlA of lineage II strains revealed a higher level of diversity. A percentage of 38.91% of strains of lineage II were categorized as group B, whereas only 3.71% of lineage I strains were categorized as group B, which is consistent with previous research results [19]. Lineage II strains exhibit the largest number of truncated InlA variants that may cause attenuated pathogenicity [46]. The association between this diversity and the complex food environment and stress exposures of this strain's source requires further investigation and confirmation.

Researchers have demonstrated that strains containing InlA surface protein with PMSCs display diminished invasive capacity within cells [1,9,21]. Nevertheless, other researchers have observed that these strains still have the capability of causing disease [19,24,47]. Therefore, varying types of PMSC in InlA may impact the virulence of *L. monocytogenes* differently. We analyzed 67 isolates of *L. monocytogenes* obtained from humans and carrying truncated InlA, with the highest frequency being PMSC type 6 (231/991). Type 6 encodes 491 amino acids and is classified under group B-4. Most of these strains belonged to CC121 (43/67) and CC9 (16/67). Previous reports indicated that ST9 and ST121 strains often possess truncated InlA and have strong adaptability to survive in food and plants, whereas they play a minor role in causing clinical cases and exhibit low invasive activity [19,33,48]. Most truncated InlA from human strains have the intact LRR region, which is the key domain contact with the receptor on the host cell. Three strains carried truncated InlA (CC3, 646aa and CC5, 605aa) belonging to lineage I. Usually, these strains belong to CC3 or CC5 presented intact InlA and were linked to listeriosis cases. Additionally, it should be emphasized that the PMSC mutation type of the three clinical isolates of ST9 was PMSC type 19. According to the study, the presence of PMSC 19 at position 976 may not impact the ability of *L. monocytogenes* to adhere to and invade epithelial cells [21]. This indicates that the existence of PMSCs may affect the ability of the strain to invade depending on their nucleotide position. It is, therefore, essential to verify not only the presence or absence of the InlA mutation but also the type of PMSC mutation. However, additional verification and studies on molecular mechanisms are required to fully understand the precise impact of different mutation types on their function.

Proteins are composed of amino acids and perform a wide range of physiological functions in the body [49]. Previous studies have identified that Phe367Ala and Tyr343Ala, both of which were shown to significantly reduce the InlA-Ecad interaction, and Tyr369Ser and the double mutant Ser192Asn-Tyr369Ser, which were shown to increase the InlA-Ecad complex formation [22]. This indicated that single amino acid changes could affect the function of InlA. Then we found that site 192, which has been identified as a single residue causing intermolecular contact, exhibited polarity change. Strains that exhibit a change from S to F at this location belong to CC8 and are situated within the LRR region. Therefore, it is hypothesized that this alteration could potentially impact the function of InlA. However, further investigation is required to determine the exact mechanism of action. The remaining 13 sites consist of one in the α-helix and the remaining in the IR, B repeat, and PA region. These sites have no direct association with the primary binding site [27,28]. Although no previous studies have reported that altering these sites could affect the function of InlA, we found that the polarity of these sites is altered, which may disrupt hydrogen bonding, ionic bonding, or hydrophobic interactions within the protein, thereby altering the folding mode and stability of the protein. This could consequently affect its function and interactions in space [50]. There are some sites that show a higher correlation with lineages and STs, which should also be focused on. Nevertheless, further studies need to be conducted to determine the exact effects.

## Figures and Tables

**Table 1:** Characteristics of truncated internalin A in clinical *Listeria monocytogenes* strains circulating in this study.

Site of Infection	B-1	B-3	B-4	B-5	Strains	PMSC Type	Lineage	Dominant ST	Dominant CC
	SS	LRR	IR	B-Repeat					
Breast milk			36		36	6	II	121	CC121
Blood	3	4	3	8	18	1, 4, 6, 12, 19, 23, 26	II (16) I (2)	9	CC9
Human		2	1	3	6	1, 5, 6, 12, 19	II (5) I (1)	9	CC9
Cerebrospinal fluid		2			2	26, 19	II	325	CC31
Vaginal swab			2		2	6	II	121	CC121
Throat swab				2	2	11, 12	II	9	CC9
Feces of a pregnant				1	1	12	II	9	CC9
Total	3	8	42	14	67				

## Data Availability

The data presented in this study are available upon request from the corresponding author.

## Supplementary Materials

The following supporting information can be downloaded at: https://www.mdpi.com/article/10.3390/microorganisms12030485/s1, Table S1: Information and 274 variable sites of 4393 *Listeria monocytogenes* genomes; Table S2: The relationship among the lineages or STs and the aa of InlA site was explored by CA.
